# *OsMKK3*, a Stress-Responsive Protein Kinase, Positively Regulates Rice Resistance to *Nilaparvata lugens* via Phytohormone Dynamics

**DOI:** 10.3390/ijms20123023

**Published:** 2019-06-20

**Authors:** Shuxing Zhou, Mengting Chen, Yuebai Zhang, Qing Gao, Ali Noman, Qi Wang, Heng Li, Lin Chen, Pengyong Zhou, Jing Lu, Yonggen Lou

**Affiliations:** 1 State Key Laboratory of Rice Biology & Ministry of Agriculture Key Lab of Agricultural Entomology, Institute of Insect Sciences, Zhejiang University, Hangzhou 310058, China; 3090100232@zju.edu.cn (S.Z.); mtchen@zju.edu.cn (M.C.); zyb601@msn.cn (Y.Z.); 11916008@zju.edu.cn (Q.G.); alinoman@gcuf.edu.pk (A.N.); q.wang302@gmail.com (Q.W.); 11616014@zju.edu.cn (H.L.); chenlin88@126.com (L.C.); zhoupy1991@163.com (P.Z.); jing_lu@zju.edu.cn (J.L.); 2 Department of Botany, Government College University, Faisalabad 38040, Pakistan

**Keywords:** kinase cascades, *Nilaparvata lugens*, plant defense, rice

## Abstract

Plants undergo several but very precise molecular, physiological, and biochemical modulations in response to biotic stresses. Mitogen-activated protein kinase (MAPK) cascades orchestrate multiple cellular processes including plant growth and development as well as plant responses against abiotic and biotic stresses. However, the role of MAPK kinases (MAPKKs/MKKs/MEKs) in the regulation of plant resistance to herbivores has not been extensively investigated. Here, we cloned a rice MKK gene, *OsMKK3*, and investigated its function. It was observed that mechanical wounding, infestation of brown planthopper (BPH) *Nilaparvata lugens*, and treatment with methyl jasmonate (MeJA) or salicylic acid (SA) could induce the expression of *OsMKK3*. The over-expression of *OsMKK3* (oe-MKK3) increased levels of jasmonic acid (JA), jasmonoyl-L-isoleucine (JA-Ile), and abscisic acid (ABA), and decreased SA levels in rice after BPH attack. Additionally, the preference for feeding and oviposition, the hatching rate of BPH eggs, and BPH nymph survival rate were significantly compromised due to over-expression of *OsMKK3*. Besides, oe-MKK3 also augmented chlorophyll content but impaired plant growth. We confirm that MKK3 plays a pivotal role in the signaling pathway. It is proposed that *OsMKK3* mediated positive regulation of rice resistance to BPH by means of herbivory-induced phytohormone dynamics.

## 1. Introduction

In natural ecosystems, plants frequently experience herbivore attacks. Plants possess complicated defense systems to respond against insect attack and protect themselves. These defense strategies include physical barriers, metabolic adjustments, signaling cascades, and expression of different herbivory-associated genes [1,2,3,4,5]. The process of plant defense involves perception of herbivores, signal transduction, reconfiguration of metabolism, and changes in phenology [6]. During defense, phytohormone-mediated signaling (e.g., with jasmonic acid (JA), salicylic acid (SA), ethylene (ET), and abscisic acid (ABA)) is critically important for increasing resistance to herbivores [7,8,9,10,11,12,13,14]. Mitogen-activated protein kinase (MAPK) cascades act upstream of hormone-mediated signaling and, therefore, pivotal in plant defense responses to herbivores [4,6,15].

MAPKs comprise 11 (I–XI) domains that have also been recorded in all serine/threonine protein kinases [16]. Phosphorylation of Thr and Tyr residues in the TxY motif of the activation loop (T-loop) activates MAPKs. This activation takes place by means of MAPK kinases (MAPKKs/MKKs/MEKs) which are also activated by MAPKK kinases (MAPKKKs/MKKKs/MEKKs) by phosphorylation of conserved Ser and/or Thr residues in their T-loop [17]. Activation of MAPK cascades results in the activation of transcription factors and enzymes, subsequently inducing downstream signaling to tackle herbivore attacks [4,15,18,19,20,21]. In *Nicotiana attenuata*, oral secretions (OS) of *Manduca sexta* rapidly induced expression of *NaSIPK* and *NaWIPK* and elicited JA, jasmonoyl-L-isoleucine (JA-Ile), SA, and ET biosynthesis [22]. A transgenic approach plays a key role in regulation of environmental stress tolerance; for example, overexpression of *OsRab7* enhanced the rice resistance to drought and heat stress [23]. Similarly, silencing of *NaMPK4* enhanced the plant resistance to *M. sexta* through a JA-independent defense pathway in *N. attenuate* [24]. Transgenic *Nicotiana tabacum* ectopically expressing *AhMPK3* increased plant resistance to *Spodoptera litura* [25]. Damage by *Nezara viridula* in *Glycine max* induced the expression of different protein kinases such as *GmMPKK1*, *GmMPK3*, and *GmMPK6* [26]. Similar MAPKs have also been recorded in *Cicer arietinum* infested by *Helicoverpa armigera*, e.g., *CaMAPK*2, *CaMAPK3*, *CaMAPK5*, *CaMAPK7*, and *CaMAPK8* [27]. In *Oryza sativa*, *OsMPK3* and *OsMPK4* were induced by *Chilo suppressalis* and positively regulated rice resistance by modulating JA signaling [28,29]. Brown planthoppers (BPHs) mediated enhancements in transcriptional levels of *OsMPK20-5*, which negatively regulated the rice resistance to BPH by modulating ethylene and nitric oxide accumulation [30]. Though the role of MAPKs in herbivore-induced plant defense has been examined extensively, we face a paucity of data with respect to the function of MKKs in the defense against herbivores.

*OsMKK3* is an ortholog of *AtMKK3* [31]. In *Arabidopsis thaliana*, MKK3 is involved in defense against pathogens, drought tolerance, JA signal transduction, and ABA and auxin responses [32,33,34,35,36]. In *Xanthomonas oryzae*-infested rice plants, OsMKK3 phosphorylated OsMPK7 and activated OsWRKY30. Furthermore, overexpression of *OsMKK3* enhanced disease resistance and up-regulated genes related to pathogenesis, maintenance of the cell wall, and metabolism [37]. Although MKK3 has been functionally characterized and evaluated against microbial attack, the impact of MKK3 in mediating plant defense responses to herbivores needs to be explored.

Rice (*O. sativa*) crop is severely damaged by various insect pests. Among them, brown planthoppers (BPHs, *Nilaparvata lugens*) destroy rice crops by phloem sucking and cause heavy losses in growth and productivity [38]. It is on record that BPH infestation in *O. sativa* induced the biosynthesis of JA, JA-Ile, SA, ABA, ET, and H_2_O_2_, which regulate the plant defense response to herbivores [12,13,39,40,41,42,43,44]. We isolated and evaluated a rice MKK3 gene, *OsMKK3*, for its probable role in rice against BPH attack. It was confirmed that *OsMKK3* was induced by mechanical damage and BPH infestation. Moreover, overexpression of *OsMKK3* enhanced rice resistance to BPH through increased JA, JA-Ile, and ABA levels as well as decreased SA levels. Taken together, our data suggest that *OsMKK3* plays a positive role of rice immune responses elicited by BPH.

## 2. Results 

### 2.1. Expression Profiles of OsMKK3

**Figure 1:**
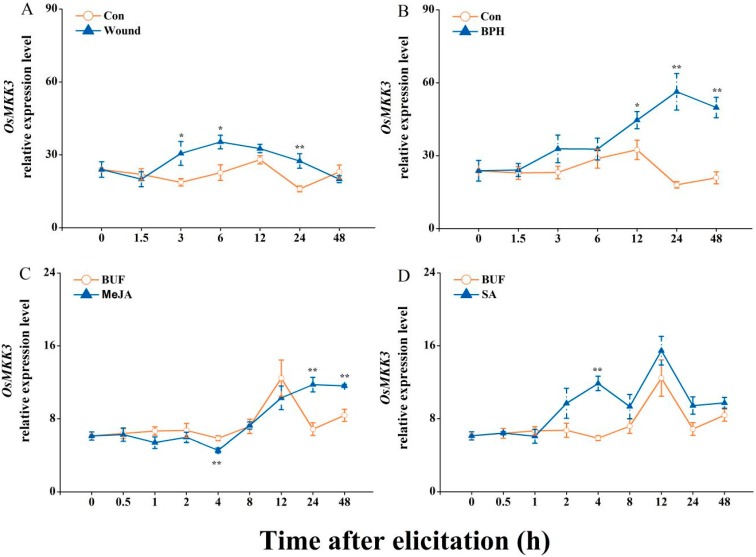
*OsMKK3* expression levels that were elicited by multiple treatments in wild-type rice. *OsMKK3* expression levels (mean ± SE, *n* = 6) in rice sheaths treated with mechanical wounding (Wound) (**A**), brown planthopper (BPH) infestation (**B**), methyl jasmonate (MeJA) (**C**), and salicylic acid (SA) (**D**) were analyzed by qRT-PCR. Con, non-treated plant, BUF, plant which absorbs buffer as mock. *(asterisk) show significant differences among control and treatments (*, *p* < 0.05; and **, *p* < 0.01, Student’s *t*-tests).

qRT-PCR analysis revealed significant differences in expression levels of *OsMKK3* in response to mechanical wounding, BPH infestation, and MeJA and SA treatments. All these treatments induced *OsMKK3* expression to different extents (Figure 1). Mechanical wounding and SA treatments rapidly induced the expression of *OsMKK3* to a maximum at 6 hours post treatment (hpt) and 4 hpt, respectively (Figure 1A,D). However, with gravid BPH females and MeJA treatments the expression of this gene was elicited at a later time, reaching a peak at 48 hpt (Figure 1B,C). These data demonstrated that *OsMKK3* might regulate rice defense in the case of BPH infestation.

### 2.2. OsMKK3 Negatively Regulates Rice Growth but Enhances Resistance to Wounding

**Figure 2:**
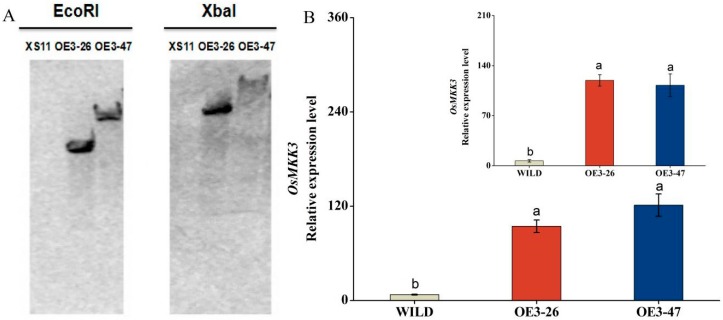
Southern blotting and *OMKK3* expression levels in oe-MKK3 (OE3-26, 47) and wild-type plants (WILD). (**A**) The DNA-hybridized band of oe-MKK3 meant a single insertion by southern blotting. (**B**) *OsMKK3* expression levels (mean ± SE, *n* = 5) in oe-MKK3 and WILD without wounding treatment were analyzed by qRT-PCR, and the insertion represented *OsMKK3* expression levels in oe-MKK3 and WILD at 8 h after wounding. Different letters represent significant differences among oe-MKK3 and wild-type plants (Duncan’s multiple range test, *p* < 0.05). oe-MKK3: transgenic lines which overexpressed *OMKK3*.

**Figure 3:**
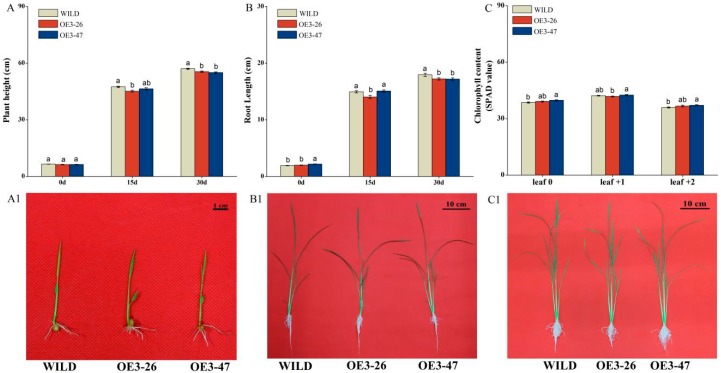
Growth attributes of *OsMKK3-*overexpressing lines (OE3-26,47) and wild-type plants (WILD). (**A**,**B**) Plant height and root length (mean ± SE, *n* = 30) of oe-MKK3 and WILD at day 0, day 15, and day 30 after transplanting to hydroponic boxes; (**A1**,**B1**,**C1**) Growth phenotypes of oe-MKK3 and WILD at day 0, day 15, and day 30 after transplanting to hydroponic boxes; (**C**) Chlorophyll content (mean ± SE, *n* = 30) of oe-MKK3 and WILD at day 30 after transplanting to hydroponic boxes; leaf 0 refers to the newest growth leaf; leaf +1 refers to the leaf which wraps leaf 0; leaf +2 refers to the leaf which wraps leaf +1. Different letters mean significant differences among oe-MKK3 and wild-type plants (Duncan’s multiple range test, *p* < 0.05).

The differences recorded in relative expression level of *OsMKK3* against JA, SA, wounding, and BPH treatments prompted us to thoroughly investigate its role in rice defense. Therefore, we obtained transgenic lines with over-expressed *OsMKK*3 and screened two T2 homozygous lines (OE3-26, 47) containing a single insertion through southern blot (Figure 2A). The transcription analysis confirmed the overexpression effect of *OsMKK3* in transgenic lines which over-expressed *OsMKK3* (oe-MKK3), exhibiting 12.9- and 16.6-fold higher expression levels of *OsMKK3* in the OE3-26 and OE3-47 lines, respectively, in comparison with wild-type plants (WILD) without wounding treatment, and 17.6-fold and 16.6-fold higher expression levels of *OsMKK3* in the OE3-26 and OE3-47 lines respectively comparing to WILD at 8 h post wounding (Figure 2B). Therefore, these two lines were selected and investigated for further research. The phenotype of wild-type plants and transgenic lines over-expressing *OsMKK3* displayed clear differences in growth parameters. Growth was significantly retarded in transgenic plants overexpressing *OsMKK3*, especially at the late growth stage, but the negative regulation of plant growth was mild. To be specific, OE3-26 and OE3-47 exhibited 2.6% and 3.5% reductions in plant height as well as 4% and 4.2% reductions in root length, respectively, in comparison to WILD at 30 days. (Figure 3A,B,A1–C1). Contrary to the phenotype, the chlorophyll content of two transgenic lines increased in comparison with wild-type plants (Figure 3C). 

### 2.3. OsMKK3 Positively Mediates Rice Defense to BPH 

**Figure 4:**
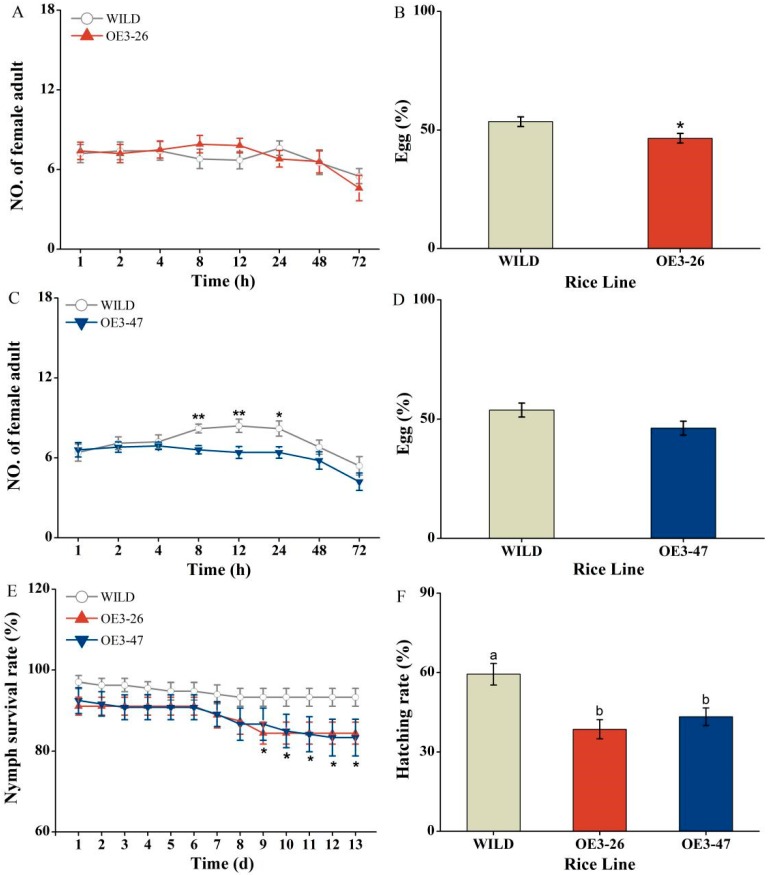
*OsMKK3* positively mediates rice defense to BPH. (**A**,**C**) Number of gravid BPH females per plant (mean ± SE, *n* = 10) on pairs of plants (wild-type plants (WILD) versus mutant lines (OE3-26 and OE3-47 line)); (**B**,**D**) BPH egg percentage per plant (mean ± SE, *n* = 10) on pairs of plants (WILD versus OE3-26 or WILD versus OE3-47) after BPH infestation for 72 h. (**E**) BPH nymph survival rate (mean ± SE, *n* = 10) on WILD, OE3-26, and OE3-47 lines; (**F**) BPH hatching rate (mean ± SE, *n* = 15) on WILD, OE3-26, and OE3-47 lines. *(asterisk) shows significant differences among WILD and oe-MKK3 (*, *p* < 0.05; and **, *p* < 0.01, Student’s *t*-tests); different letters represent significant differences among WILD and oe-MKK3. (Duncan’s multiple range test, *p* < 0.05).

As mentioned earlier, we recorded an abundance of *OsMKK3* transcripts after BPH infestation; this instigated us to validate whether overexpression of *OsMKK3* could influence the defense of transgenic rice plants under BPH attack. As shown in Figure 4A–D, we found that gravid BPH females had a preference for feeding and oviposition on wild-type plants (WILD) as compared with transgenic lines (OE3-26, 47). To be specific, OE3-26 showed a decrease in egg percentage by 7% compared with WILD but had no obvious effect on BPH feeding. In contrast, OE3-47 exhibited no significant reduction in BPH oviposition but decreased BPH feeding comparing with WILD. On the other hand, BPH preference of OE3-26 and OE3-47 resulted in a significantly reduced hatching rate of eggs, i.e., 35% and 27%, respectively, compared with WILD (Figure 4F). Additionally, BPH nymph survival rates on oe-MKK3 were decreased by about 10% in comparison with WILD, demonstrating that over-accumulation of *OsMKK3* enhanced rice resistance to BPH (Figure 4E).

### 2.4. Overexpressed OsMKK3 Alters the Accumulation of JA, JA-Ile, ABA, and SA but Not Ethylene and H_2_O_2_ in Response to BPH Infestation

**Figure 5:**
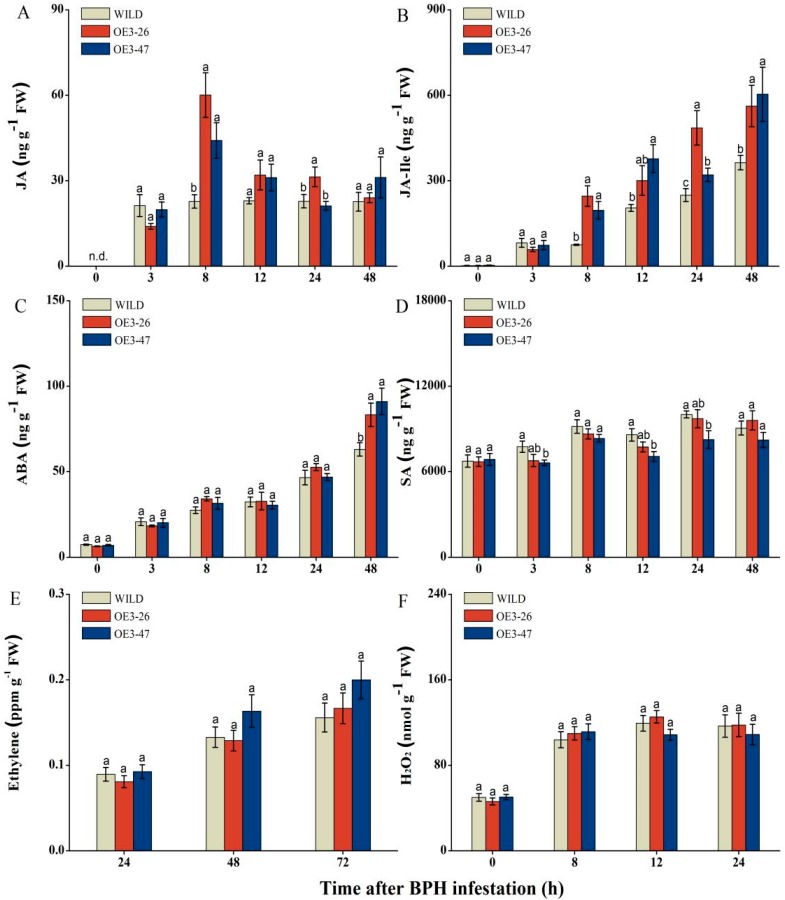
*OsMKK3* mediates the levels of jasmonic acid (JA), jasmonoyl-L-isoleucine (JA-Ile), abscisic acid (ABA), and salicylic acid (SA), but not ethylene and H_2_O_2_ in response to BPH. Levels (mean ± SE, *n* = 6) of JA (**A**), JA-Ile (**B**), ABA **(C)**, SA (**D**), ethylene (**E**), and H_2_O_2_ (**F**) in overexpressed lines (OE3-26, 47) and wild-type plants (WILD) after infestation of 15 gravid BPH females. Different letters refer to significant differences among WILD, OE3-26, and OE3-47. (Duncan’s multiple range test, *p* < 0.05).

Numerous reports have suggested that JA, JA-Ile, ABA, SA, ethylene, and H_2_O_2_ play vital roles in rice resistance to BPH [12,13,39,40,41,42,43,44,45,46,47,48]. Therefore, we targeted phytohormone pathways to determine whether the enhanced resistance to BPH in two *OsMKK3*-overexpressing lines related with them or not. We examined elicitation of JA and JA-Ile by gravid BPH females in both transgenic lines (OE3-26, 47) and wild-type plants (WILD). Under BPH infestation, the levels of these two signals were much higher in oe-MKK3 lines than WILD. At 8 hours post BPH infestation (hpi), the JA levels exhibited remarkable differences between oe-MKK3 and WILD; meanwhile, the significant difference for JA-Ile lasted from 8 hpi to 48 hpi. The levels of these two signals showed the most remarkable difference at 8 hpi; to be specific, OE3-26 and OE3-47 exhibited 2.6- and 1.9-fold higher levels of JA as well as 3.3- and 2.6-fold higher levels of JA-Ile, respectively, in comparison with WILD at 8 hpi (Figure 5A,B). Moreover, just like JA and JA-Ile, an obvious high induction amplitude was observed for ABA levels following BPH attack in transgenic lines at 48 hpi, exhibiting 1.3- and 1.4-fold higher levels of ABA in OE3-26 and OE3-47 separately compared with WILD (Figure 5C). On the contrary, the SA levels in transgenic plants decreased slightly at 3 h, 12 h, and 24 h after infestation compared with wild-type plants; moreover, the accumulation of SA in OE3-26 and OE3-47 decreased 12.6% and 14.6%, 9.9% and 17.7%, and 3% and 17.7% at 3 hpi, 12 hpi, and 24 hpi, respectively. However, just the SA level in OE3-47 declined significantly. (Figure 5D). Although BPH induced ethylene and H_2_O_2_ accumulation, the difference was non-significant among transgenic and wild-type plants (Figure 5E,F). These results imply that the *OsMKK3* overexpression mediating rice defense against BPH infestation was JA-, JA-Ile-, and ABA signal pathway-dependent. It was confirmed that overexpression of *OsMKK3* significantly increased their biosynthesis and decreased SA production, but did not influence the accumulation of ethylene and H_2_O_2_ in BPH-infested plants. 

## 3. Discussion

In this study, BPH-induced overexpression of *OsMKK3* increased the rice resistance to BPH (Figure 4) by enhancing the accumulation of BPH-induced JA, JA-Ile, and ABA but weakening the level of SA slightly (Figure 5A–D). Additionally, oe-MKK3 impaired plant growth slightly but increased the content of chlorophyll (Figure 3). These findings indicated positive regulation of rice resistance to BPH by *OsMKK3* but negatively regulated plant growth.

Like other MAPKs, MKK3 participates in various plant responses. In *Arabidopsis*, *AtMKK3* could be induced by treatments of JA, MeJA, wounding, cold, NaCl, blue light, and ABA as well as by pathogen infestation [32,34,35,49,50,51,52]. Similarly, in rice *X. oryzae* infestation and H_2_O_2_ production resulted in strong induction of *OsMKK3*; however, the expression of *OsMKK3* was induced slightly at 2 h post SA treatment [37]. The present study confirms the earlier reports. Our results demonstrated that BPH infestation, mechanical wounding, and treatment with MeJA and SA could elicit the expression of *OsMKK3* (Figure 1). This analysis provides evidence for a significant involvement of *OsMKK3* in biotic- and abiotic stress-induced defense. 

In the light of our results, it is worth discussing the interesting facts revealed by other authors. In line with previous studies, we confirmed that MKK3 plays a pivotal role in signaling pathways. For instance, in *Arabidopsis*, JA activated the MKK3-MPK6 cascade, which negatively regulated MYC and positively regulated the expression of PDF1.2 but not ERF1. Furthermore, interestingly, this cascade did not affect ethylene accumulation [32]. Likewise, in the necrotrophic pathogen infestation of *Arabidopsis*, activated MKK3 triggered JA accumulation that led to activation of defense responses [50]. A similar pattern of results was obtained in an *Arabidopsis*-activated MKK3–MPK6 cascade, showing a crucial role in JA signaling for enhancing the resistance to *Salmonella* [53]. These facts represent interconnected elements of plant defenses i.e., protein kinases and phytohormones. Our findings regarding JA and JA-Ile involvement (increased levels) and unaffected ET levels are supported by this evidence. Uninfluenced H_2_O_2_ has also been observed in *OsMKK3* overexpression lines during pathogen infestation. Here, the discrepancy of H_2_O_2_ may result from different defensive mechanisms which are triggered by herbivores and pathogens in plants. ABA treatment activated MAPKKK17/18, which participated in ABA signaling through the MKK3-MPK1/2/7/14 cascade [35,54,55]. In addition to the MKK3–MPK7 cascade response to H_2_O_2_ in *Arabidopsis* [34], OsMKK3 interacted with OsMPK7 in response to H_2_O_2_ and positively regulated resistance to *X. oryzae* in rice [37]. This points out that instead of signaling interaction with phytohormones only, protein kinases can also interact with each other to confer a robust defense response. Such an interaction may require other molecules e.g., H_2_O_2_, as a trigger. Accumulated JA, JA-Ile and ABA in oe-MKK3 plants is in accordance with the function of MKK3 in *A. thalianas*, which activates its downstream MAPKs (MPK1/2/6/7/14) to regulate this signaling. Numerous studies have reported that this accumulation of JA and ABA is supported by the fact that JA and ABA interact synergistically, whereas they have antagonistic interactions with SA in plant defense [7,10,56]. Numerous studies have revealed that some MAPKs acted as negative regulators in SA signaling in plant defense; for example, in *Arabidopsis*, *AtMPK3* and *AtMPK4* negatively regulated flg22-induced SA accumulation [57], and in *N. tabacum*, *NtMPK3* and *NtMPK6* suppressed wound-induced SA accumulation [58]. On the contrary, several MAPKs positively regulate SA signaling; for instance, overexpression of *OsMPK6* increased *X. oryzae-*induced SA accumulation and overexpression of *OsMPK4* elevated *C. suppressalis-*induced SA levels in rice [29,59]. Therefore we attribute slight decrease in SA among overexpression *OsMKK3* plants to the negative regulation of SA via its downstream MAPKs or the antagonistic interaction between JA and SA. Additionally, our results indicated that SA elicited the expression of *OsMKK3* which was a negative regulator of SA. Similarly, *AtMPK3* could be induced by SA but negatively regulated SA accumulation in *Arabidopsis* [57,60]. We speculate *OsMKK3* might have a negative feedback to SA via its downstream MAPKs. JA and ABA signaling pathways positively mediate the rice resistance to BPH whereas SA signaling pathway is opposite [39,45,46,61,62,63,64]. Guo et al. and Liu et al. explained that exogenous MeJA decreased BPH survival rate in rice and exogenous ABA hampered BPH feeding and fecundity but enhanced rice resistance through increased callose deposition [45,46]. Therefore, overexpression of *OsMKK3* enhanced the rice resistance to BPH may result from the increased biosynthesis of JA, JA-Ile as well as ABA and decreased SA. However, some studies report that SA signaling pathway is a positive regulator, while the JA signaling pathway is a negative regulator in rice resistance to BPH [42,43,44,47,65,66]. This conflict may be due to the fact that when plants are attacked by BPH, they will alter signal molecules differentially according to different conditions. Additionally, Os*MKK3* plays a crucial role in the defense of plants to biotic stress. Overexpression of *OsMKK3* enhanced rice resistance to *X. oryzae* through the OsMKK3–OsMPK7–OsWRKY30 pathway, which induced up-regulated genes related with pathogenesis, maintenance of the cell wall, and metabolism [37]. Moreover, for other MAPKs it has been reported that they participate in herbivore-induced defense. For example, *OsMPK3* and *OsMPK4* positively regulated rice resistance to *C. suppressalis* by JA and trypsin proteinase inhibitors, while *OsMPK20-5* negatively regulated rice resistance to BPH by ethylene and NO [28,29,30]. *NaWIPK* and *NaSIPK* act as positive regulators of *M. sexta*-induced expression levels of *NaWRKY6* and CDPKs as well as accumulation of phytohormones (JA, SA) and defense metabolites (nicotine, trypsin proteinase inhibitors, caffeoylputrescine, and volatiles) [22,67]. Taken together, we speculate that when rice is attacked by BPH, *OsMKK3* may activate downstream MAPKs and WRKYs, causing accumulation of phytohormones and defense metabolites to enhance rice resistance to BPH. However, the mechanistic details about how *OsMKK3* influenced signaling pathways in resisting BPH are largely unknown and should be studied further. 

Despite the positive regulation of rice defense by *OsMKK3*, we noticed its involvement in plant growth and chlorophyll content in this study. Overexpression of *OsMKK3* impaired plant growth compared with wild-type plants, i.e., shortened plant height and root length, and increased chlorophyll content (Figure 3). Such growth retardation in mutants is in line with previous reports. For example, MKK3 negatively influenced plant growth which indicated that MKK3 mediated overexpression *AtMAPKKK17/18* attributing impaired *Arabidopsis* growth such as decreased plant weight and rosette diameter [54,55]. Meanwhile, Enders indicated that the MKK3–MPK1 cascade pathway has a negative regulation on auxin-dependent cell growth in *Arabidopsis* [36]. Additionally, other MAPKs are also involved in auxin signaling. For example, *AtMPK3*, *AtMPK6*, and *AtMPK12* act as negative regulators in auxin signaling [68,69]. As we know, auxin plays a vital role in plant growth and development, so we attribute the growth retardation in oe-MKK3 to repressed auxin signaling. However, our observation regarding increased chlorophyll content is contrary to the available information. A comparison of results shows that overexpression of *AtMAPKKK18* activated MKK3 to lower the chlorophyll content in *Arabidopsis* [54]. A difference between these results can be attributable to dissimilar effect of MAPK cascades in different plant species. 

## 4. Materials and Methods

### 4.1. Plant Growth

Seeds of wild-type rice (Xiushui 11, WILD) and two overexpressed *OsMKK3* transgenic lines (oe-MKK3, OE3-26, and OE3-47) were pre-germinated and grown in an incubator at 28 ± 2 °C with a 14-h light period. Seedlings (10 days) were shifted to hydroponic boxes (20 L) containing nutrient media and placed in growth room (28 ± 2 °C, 14-h light phase, 55–65% relative humidity). After 25–30 days, seedlings of good health and with consistent growth were transplanted in 300-mL hydroponic jars with nutrient solution. Four days later, plants were used for experiments.

### 4.2. Insects

A BPH colony was collected from rice fields in Zhejiang province and reared on BPH-susceptible rice variety TN1 under favorable conditions (28 ± 2 °C, 14-h light phase, 55–65% RH).

### 4.3. Isolation and Characterization of OsMKK3 cDNA

PCR was used to amplify the cDNA of *OsMKK3*. The forward and reverse primers MKK3-F (5′-AGGTCCGCCCTATCCAGC-3′) and MKK3-R (5′-TAACTGCAAACAAAGCAATCCCTAG-3′) were designed according to the *OsMKK3* (Os06g27890) sequence. In pMD19-T vector (TaKaRa, Tokyo, Japan), PCR products were cloned and sequenced.

### 4.4. Generation of Transgenic Plants

To prepare an overexpression construct, *OsMKK3* full-length cDNA was cloned with pCAMBIA-1301 vector. Rice variety Xiushui 11 was transformed with this vector by using *Agrobacterium*. Using GUS staining and southern blot, homozygous T2 transgenic plants were screened and confirmed [40]. T2 homozygous lines i.e., OE3-26, OE3-47, were screened and adopted for further experimentation.

### 4.5. Plant Treatments

For mechanical injury, 2-cm-long basal portions of leaf sheaths were separately wounded for 200 pricks with a needle. Non-wounded plants were considered control for comparisons (Con). For recording responses to BPH attack, each plant covered with a glass cylinder was individually attacked with 15 gravid female BPHs. Non-infested plants were considered as the control for comparisons (Con). For the treatment of methyl jasmonate (MeJA) and SA, each plant absorbed the nutrient solution which was mixed with the ethyl alcohol (70%), dissolving MeJA (100 μM) and SA (100 μM), respectively. Plants supplied with ethyl alcohol (70%) only were used as controls (BUF). The experiment was performed with six replications.

### 4.6. qRT-PCR

The SV Total RNA Isolation System was used to isolate plant total RNA, with subsequent reverse transcription to cDNA by CFX96 Real-Time system (Bio-RAD, Hercules, CA, USA). We performed qRT-PCR for *OsMKK3* (Os06g27890) together with an internal standard *OsACTIN* (Os03g50885) for normalizing concentrations of all the sample cDNA. Details on *OsMKK3* and *OsACTIN* primers and probes are provided in Table 1.

### 4.7. Measurement of Plant Growth Parameters

The plant height and root length were measured with the help of a ruler. Plant height and root length of transgenic plants (OE3-26, OE3-47) and wild-type plants (WILD) were measured at 0, 15 and 30 days (d) after transfer to hydroponic boxes. For the determination of chlorophyll content, 30-days-old three-identical-position leaves on each plant were selected. Chlorophyll was measured by the chlorophyll meter SPAD-502 Plus. Thirty replications of each rice line at each growth stage were performed for this experiment. 

### 4.8. BPH Performance Measurement

To observe the impact of overexpressing *OsMKK3* on BPH preference for feeding and oviposition, individual plants (OE3-26 or OE3-47, versus WILD) were confined in a glass jar. Gravid females BPH (15) were released in every jar and number of females BPH was recorded on every plant after 1, 2, 4, 8, 12, 24, 48, and 72 h. After 72 h of female BPH infestation, every plant was dissected and observed under microscope for recording the number of eggs. Data were collected in 10 replicates.

Survival rates and developmental duration were recorded to assess impact of transgenic plants on BPH nymphs. Fifteen BPH nymphs (newly hatched) were released on each plant (WILD, OE3-26 and OE3-47) confined with a glass cylinder. Until their final emergence as adults, all the surviving nymphs were counted and recorded each day. Post-emergence, the BPH nymph survival rate and developmental duration were calculated for each plant. The experiment of each rice line was replicated 10 times.

To compare the difference of the hatching rate of BPH eggs between transgenic plants and wild-type plants, 10 gravid female BPHs were allowed to lay eggs on each plant for 24 h. The numbers of BPH nymphs (newly-hatched) on transgenic plants (OE3-26, OE3-47) and wild-type plants (WILD) were noted until the total absence of nymphs. Plant was dissected under microscope and the number of unhatched eggs was recorded to evaluate the hatching rate. The experiments for each rice line were replicated 15 times.

### 4.9. JA, JA-Ile, ABA, and SA Analysis

Every plant was infested by 15 gravid BPH females confined in a covered glass jar. We harvested the sheaths of non-infested plants as 0 hpi. The sheaths were collected at 3, 8, 12, 24, and 48 hpi. After grinding the samples in liquid nitrogen, we extracted the JA, JA-Ile, and ABA as well as SA of samples by ethyl acetate, which contained their labelled internal standards, through vortex, centrifugation, and concentration. Then we analyzed their contents by an HPLC-MS-MS according to Lu et al. [70]. Six replications of each rice line at each time point were performed for this experiment.

### 4.10. Ethylene Analysis

Every plant with a closed glass jar received 15 gravid BPH females. The production of ethylene was measured at 24, 48, and 72 hpi by GC-MS based on the method of Lu et al. [71]. Ten replications of each rice line at each time point were performed for this experiment. 

### 4.11. Hydrogen Peroxide Analysis

Every plant was infested by 15 gravid BPH females covered with a glass cylinder. We harvested the sheaths of non-infested and infested plants at 0 hpi. At 8, 12, and 24 hpi, we ground the sheaths in liquid nitrogen and extracted the H_2_O_2_ of samples by cold water through vortex and centrifugation. Then, we determined H_2_O_2_ concentrations of all the samples by the Amplex^®^ Red Hydrogen Peroxide/Peroxidase Assay Kit (Invitrogen, Eugene, OR, USA). A standard curve (0, 0.625, 1.25, 2.5, 5, 10, 20, 40 nmol/mL) was used to normalize the H_2_O_2_ concentration of every sample. Six replications of each rice line at each time point were performed for this experiment.

### 4.12. Data Analysis

We analyzed the differences with respect to *OsMKK3* expression level, plant height, root length, chlorophyll, and BPH hatching rate as well as the level of phytohormones and H_2_O_2_ between oe-MKK3 and wild-type plants by one-way ANOVA with PASW statistics software. Meanwhile, we also analyzed the differences in means of *OsMKK3* expression levels between controls and treated plants by Student’s *t*-test with PASW statistics software.

## 5. Conclusions

We conclude that *OsMKK3* is a positive regulator of BPH-induced rice defense through the JA, ABA, and SA signaling pathways, whereas it is a negative regulator of rice growth. This provides new evidence for the crucial role of MAPKs in plant defense against herbivores as well as a theoretical basis for controlling pests in the fields.

## Figures and Tables

**Table 1:** qRT-PCR primers and probes.

GENE	LOCUS	F-PRIMER (5′-3′)	R-PRIMER (5′-3′)	PROBE
*OsACTIN*	Os03g50885	TGGACAGGTTATCACCATTGGT	CCGCAGCTTCCATTCCTATG	HEX-CGTTTCCGCTGCCCTGAGGTCC-BHQ1
*OsMKK3*	Os06g27890	TTTAGTTGAATTCCAGGGTGCA	AAGGACCCACCATCCATGTATT	FAM-TTCTGGACAAATAAGCATCGCCCT-BHQ2
